# Supplementary material for: Invariant Natural Killer T-Cells and Total CD1d Restricted Cells Differentially Influence Lipid Metabolism and Atherosclerosis in Low Density Receptor Deficient Mice
Source: Int J Mol Sci. 2019 Sep 14;20(18):4566. doi: 10.3390/ijms20184566 (PMC6770011; doi:10.3390/ijms20184566)
Supplement: Supplementary file 1 [file ijms-20-04566-s001.zip › ijms-573895-SI.pdf]

SUPPLEMENTAL MATERIAL

Supplemental Methods

Transcript levels in spleen and liver. Spleens and livers were obtained from the mice prior to vascular perfusion and a portion flash frozen in liquid nitrogen. For semi-quantitative PCR, RNA was isolated using Qiagen RNeasy Mini spin column kit. Reverse transcription was carried out using oligo dT primers (Invitrogen) and either the Transcriptor RT system (Roche) or the Sensiscript RT (Qiagen). For each gene screened, serial dilutions of template cDNA were assayed to define the linear range of amplification for subsequent polymerase chain reaction using FastStart Taq High Fidelity PCR system (Roche). The PCR products were run on ethidium bromide stained agarose gels in TBE. Gel images were acquired with an Alpha Innotech FluorChem 8800 Image Detection System and target bands quantified using AlphEaseFC™ analysis software and normalized to GAPDH. For real time PCR, RNA was isolated using Trizol and cDNA was prepared using Superscript III (Invitrogen). Real time PCR using SYBR green was performed using RT PCR primer pairs from SABiosciences and ABI StepOne Real Time PCR machine. HPRT was used as the endogenous control.

Supplemental Figures and Tables

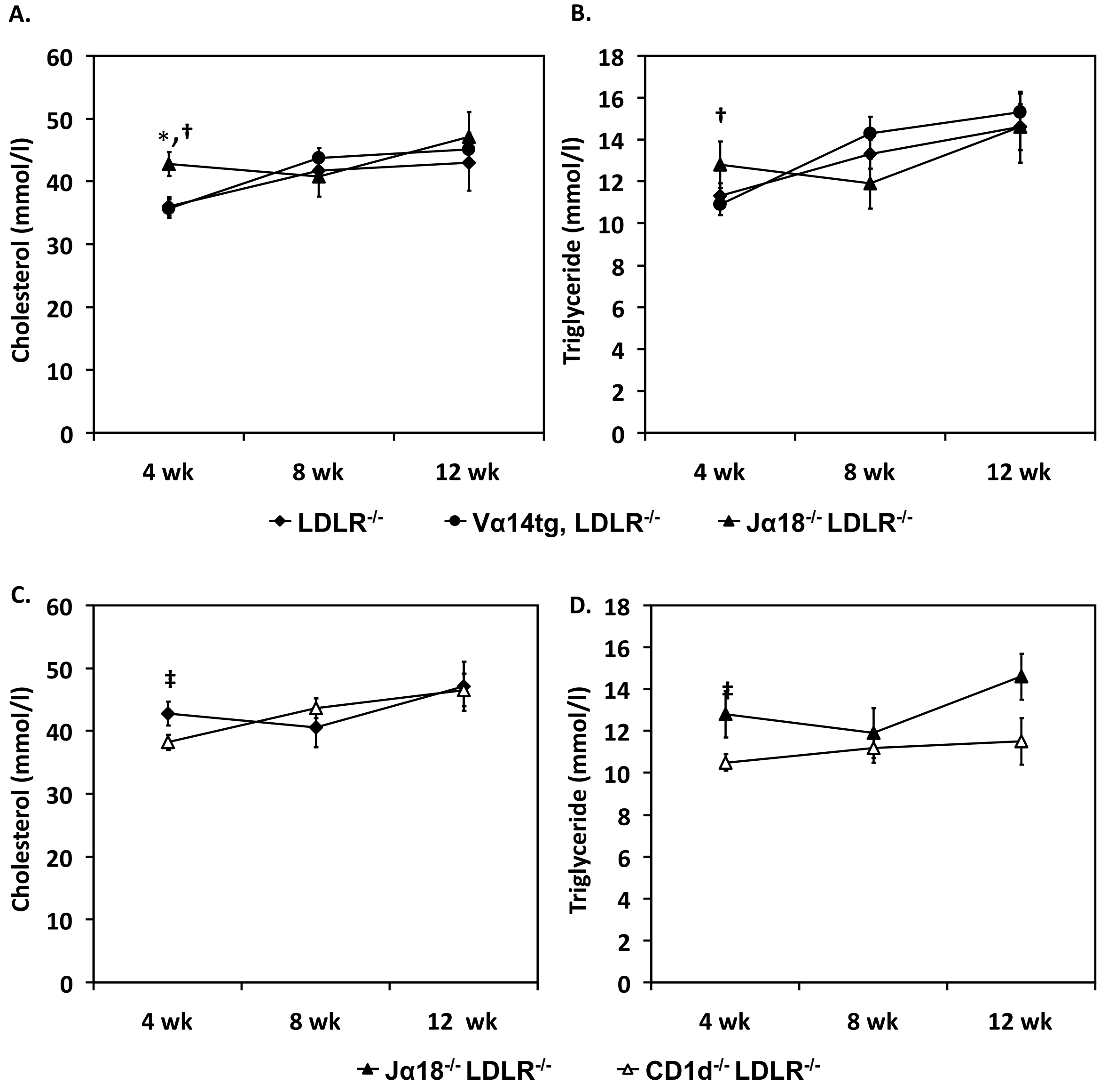

Figure S1: Plasma Lipid Levels in Male Mice. Plasma cholesterol (A, C) and triglyceride (B-D) in 4 hour fasted plasma was measured every 4 weeks. Significance $p<0.005$: * $LDLR^{-/-}$ vs. $J\alpha18^{-/-}LDLR^{-/-}$; † $V\alpha14tg, LDLR^{-/-}$ vs. $J\alpha18^{-/-}LDLR^{-/-}$; ‡$CD1d^{-/-}LDLR^{-/-}$ vs. $J\alpha18^{-/-}LDLR^{-/-}$. For 4 weeks: n= 26-44. For 8 weeks; n = 11-30. For 12 weeks; n = 9-13.

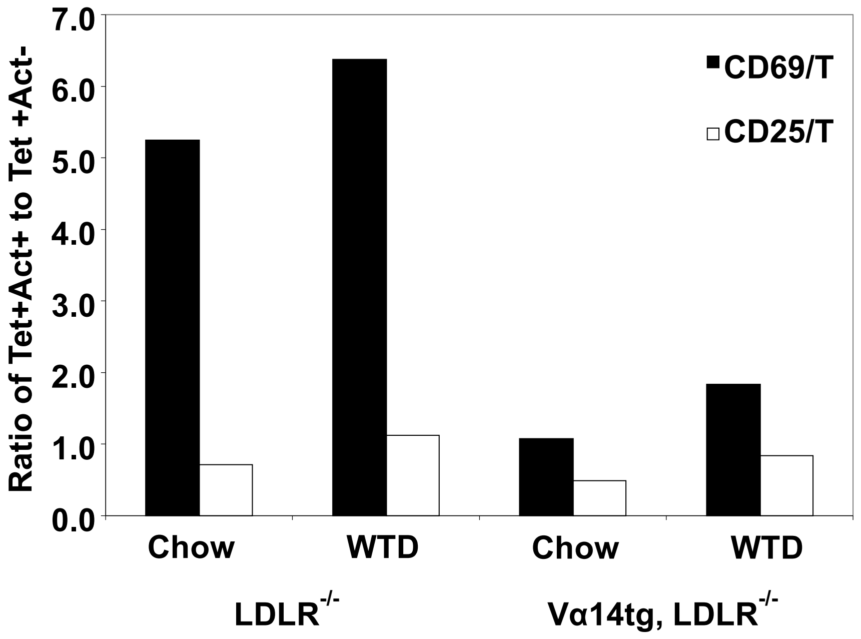

Figure S2: Activation ratios of αGalCer^{+} cells in livers of LDLR$^{-/-}$ and Vα14tg, LDLR$^{-/-}$ mice. Liver lymphocytes from mice fed WTD for 3 weeks were stained with αGalCer-loaded CD1d-Tetramer and either CD69 or CD25 for FACS analysis (n=2-4 per group).

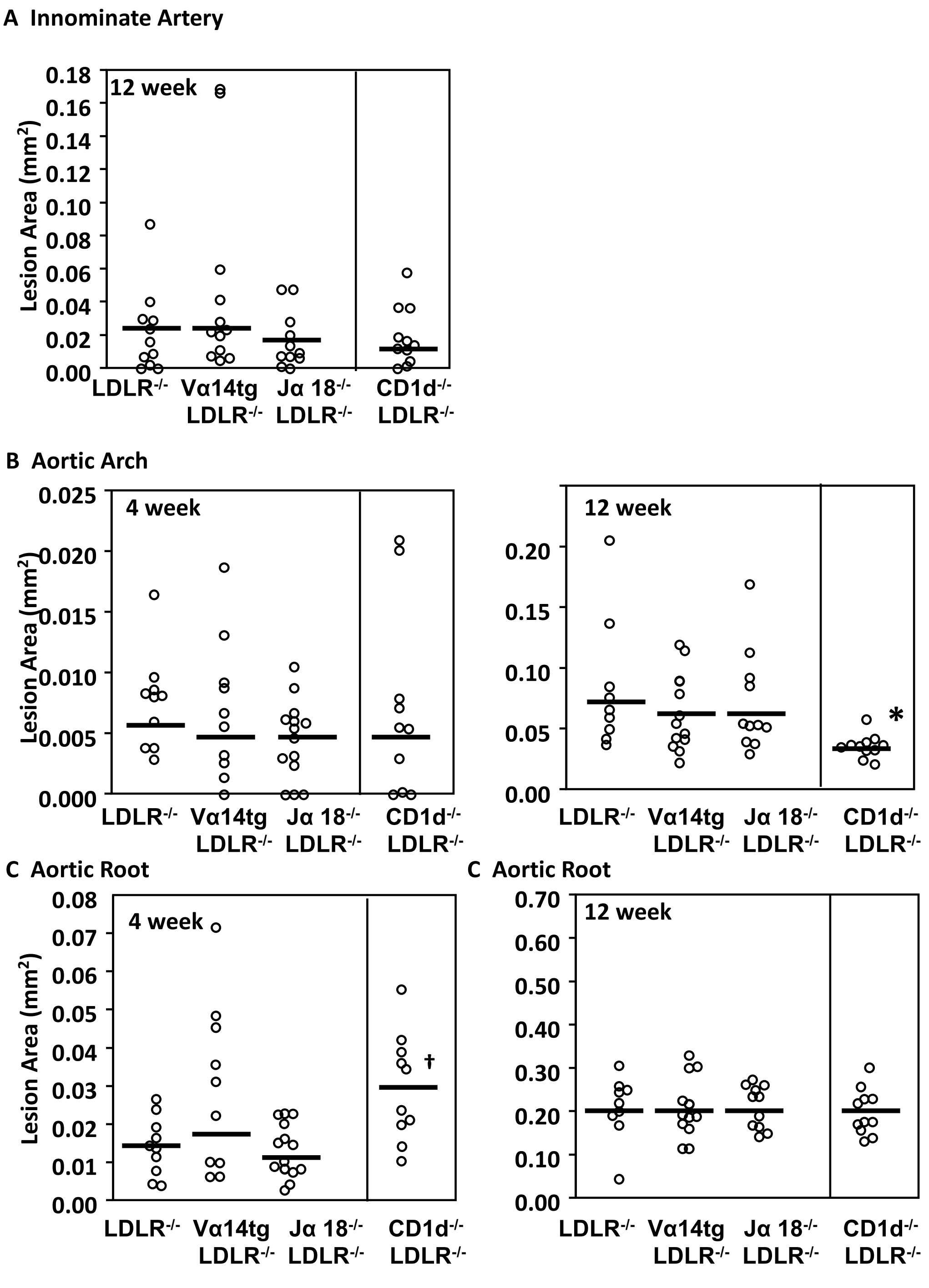

Figure S3: Atherosclerosis in male mice. Atherosclerosis was examined in the innominate artery, aortic arch, and aortic root after 4 and 12 weeks on WTD. Significant difference between Jα18$^{-/-}$LDLR$^{-/-}$ and CD1d$^{-/-}$LDLR$^{-/-}$ mice, $^{\dagger}p<0.01$, $*p<0.05$

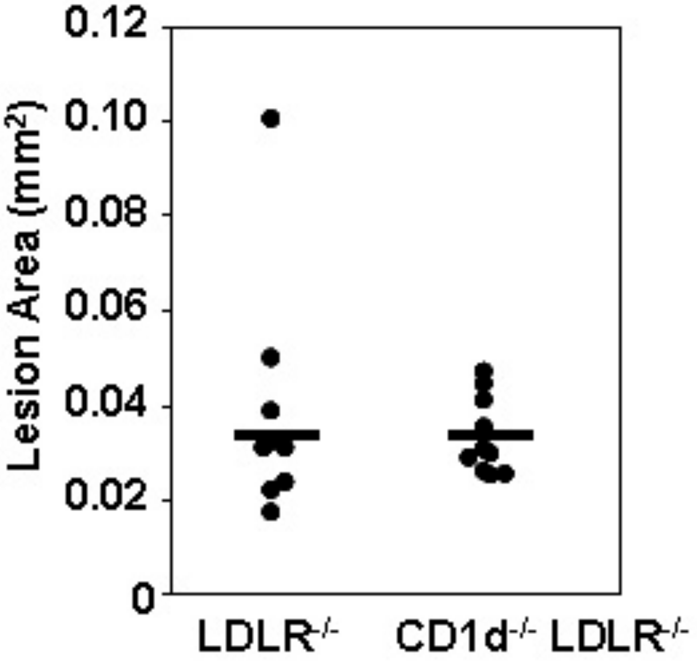

Figure S4: Aortic root atherosclerosis. LDLR$^{-/-}$ and CD1d$^{-/-}$LDLR$^{-/-}$ mice were fed a diet a diet containing 1.25% cholesterol, 21% fat (Research Diets D12108) for 4 weeks. N = 10 for each strain.

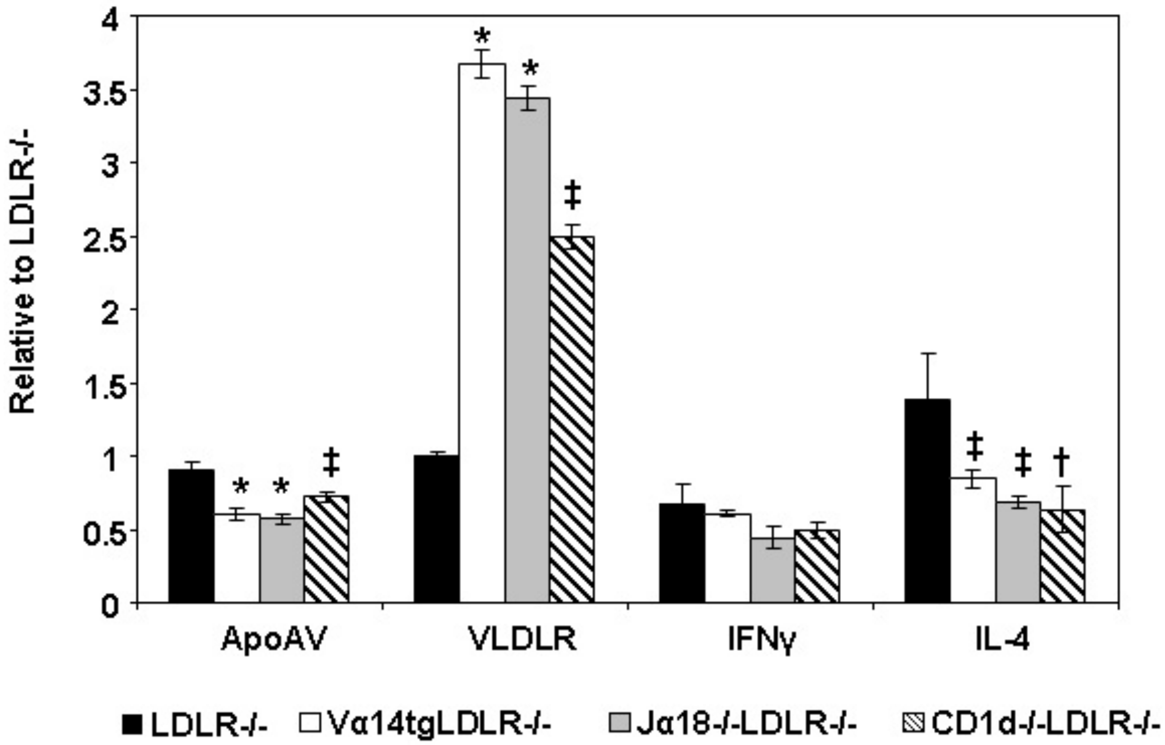

Figure S5: Relative hepatic mRNA levels. Livers from female mice fed Western type diet for 12 weeks were analyzed for the indicated mRNA by real time PCR. Significance versus LDLR$^{-/-}$ mice: * $p<0.005$; $^{\dagger}p<0.01$, $^{\ddagger}p<0.05$. n = 4 per strain.

Table S1: Lesion grading scale

Murine Atherosclerosis Lesion Grading Scale	
**0**	**Normal Vessel**<br>Tight packed lamellar vascular smooth muscle cells in the medial layer (~5 layers), no subendothelial space evident.
**1**	**Early Changes**<br>Ballooning of vascular smooth muscle cells, lipid retention in medial layer, but no foam cell accumulation or luminal obstruction.
**2**	**Fatty Streak**<br>Initial accumulation of either individual or small clusters of macrophage foam cells within the subendothelial space, mild protrusion into the lumen, no single confluent lesion yet.
**3**	**Intimal Xanthomata**<br>Accumulation of significant macrophage foam cells in a given vascular site, confluent lesion, mild luminal obstruction, no migration or involvement of vascular smooth muscle cells apparent.
**4**	**Early Atherosclerotic Lesion**<br>Like an intimal xanthomata, but demonstrating the first signs of vascular smooth muscle cell involvement in the subendothelial space forming the beginnings of a fibrous cap.
**5**	**Progressing Atherosclerotic Lesion**<br>Early evidence of macrophage apoptosis at the medial borders of the lesion, more developed fibrous cap.
**6**	**Mature Atherosclerotic Plaque**<br>Significant fibrous cap and extracellular matrix evident, large acellular lipid filled necrotic core.
**7**	**Advanced Atherosclerotic Plaque**<br>A large mature atherosclerotic plaque with signs of multi-lamellar lesion formation (2° or 3° lesions developing atop of an existing fibrous cap).
**8**	**Complex Atherosclerotic Plaque**<br>A large advanced atherosclerotic plaque with evidence of any of the following: intraplaque hemorrhage, calcification, ossification, chondrification, or aneurysmal breach of the underlying medial layers.
**9**	**Ruptured Atherosclerotic Plaque**<br>A large advanced atherosclerotic plaque with a breach of the luminal surface of the fibrous cap leading to a luminal atherothrombosis.
**10**	**Fibrotic Atherosclerotic Plaque**<br>Observed in the regression of complex atherosclerotic plaques, significant fibro-proliferative smooth muscle cell and extracellular matrix components with a paucity of macrophage foam cells or extracellular lipid pools.

Gene	Amplicon	Primers
CD3ε	350 bp	For: 5' CAT CTG TAT CAC TCT GGG CTT GCT<br>Rev: 5' AAG GTT CTA GGA CAC GTG TTC ACC
TNFα	603 bp	For: 5' CTC AGC CTC TTC TCA TTC CTG CTT<br>Rev: 5' AAT GAC TCC AAA GTA GAC CTG CCC
IL-10	458 bp	For: 5' ACT GCT ATG CTG CCT GCT CTT ACT<br>Rev: 5' TGG CCT TGT AGA CAC CTT GGT CTT
TGF-β1	695 bp	For: 5' TAA AGA GGT CAC CCG CGT GCT AAT<br>Rev: 5' TGT ACT GTG TGT CCA GGC TCC AAA
SR-B1	239 bp	For: 5' AGT CTC GGC TTC TGT CAT CTC TGT<br>Rev: 5' TCG GGT CTA TGC GGA CAT TCT TGA
ABCA1	795 bp	For: 5' ATT ACC TCC TTC CTG GGC CAC AAT<br>Rev: 5' AGA CAC GTG CTT CCT GAT GAG GTT
CD36	491 bp	For: 5' CAG CCC AAT GGA GCC ATC<br>Rev: 5' AAC ACA GCC TAG ATA GAC CTG C
CD69	604 bp	For: 5' GCA AGT ACA ATT GCC CAG GCT TGT<br>Rev: 5' AGT TTC AGC TCC TGG TCA AGG GAT
LRP	377 bp	For: 5' GCA GAT GGC AAG ACG TGC AAA GAT<br>Rev: 5' TGG TAT GCT CAT CCA CAA AGC CCT
Fatty acid synthase (FAS)	569 bp	For: 5' CCT TCT GGT GTG CAA CTG TGC ATT<br>Rev: 5' TCA CGA GGT CAT GCT TTA GCA CCT
MTTP	~700 bp	For: 5' GGA CTT TTT GGA TTT CAA AAG TGA C<br>Rev: 5' GGA GAA ACG GTC ATA ATT GTG
SREBP1	491 bp	For: 5' ACA GCT GTG AAG ACA GAT GCA GGA<br>Rev: 5' ACT TCG GGT TTC ATG CCC TCC ATA
apoA1	886 bp	For: 5' CTG TCG GAG AGC TCC GGG GAG GTC AC<br>Rev: 5' TTT ATT GTA AGA AAG CCA ATG CGG
apoA2	209 bp	For: 5' TGT AGC CTG GAA GGA GCT TTG GTT<br>Rev: 5' AGA CTA GTT CCT GCT GAC CTG ACA
apoA5	606 bp	For: 5' AAC AGT TGG AGC AAA GGC GTG ATG<br>Rev: 5' ACG TGT GAG TTT GTG GGA CAG AGT
apoC1	309 bp	For: 5' TGT CCT GAT TGT GGT CGT AGC CAT<br>Rev: 5' TGA TGA AGA GGG ACC TGG CAC ATT
apoC2	258 bp	For: 5' AGA GCT ATA AAG CCT GCC AAC CCA<br>Rev: 5' TCT GGT ACA GGT CTT TGG CAA CCT
apoC3	232 bp	For: 5' TAC AGG GCT ACA TGG AAC AAG CCT<br>Rev: 5' ACA GGC ACA TCT GCA ACA CAG AAG
apoE	707 bp	For: 5' TGA CAG ATC AGC TCG AGT GGC AAA<br>Rev: 5' TTG CTG GGT CTG TTC CTC CAT CTT
GAPDH	438 bp	For: 5' TGC CAT TTG CAG TGG CAA AGT GG<br>Rev: 5' TTG TCA TGG ATG ACC TTG GCC AGG

Table S2. Genes examined by semi-quantitative PCR in the liver of Vα14tg LDLR$^{-/-}$ and CD1d$^{-/-}$LDLR$^{-/-}$ mice for which no differences were observed. n = 4 mice per strain.
